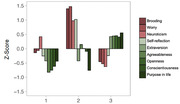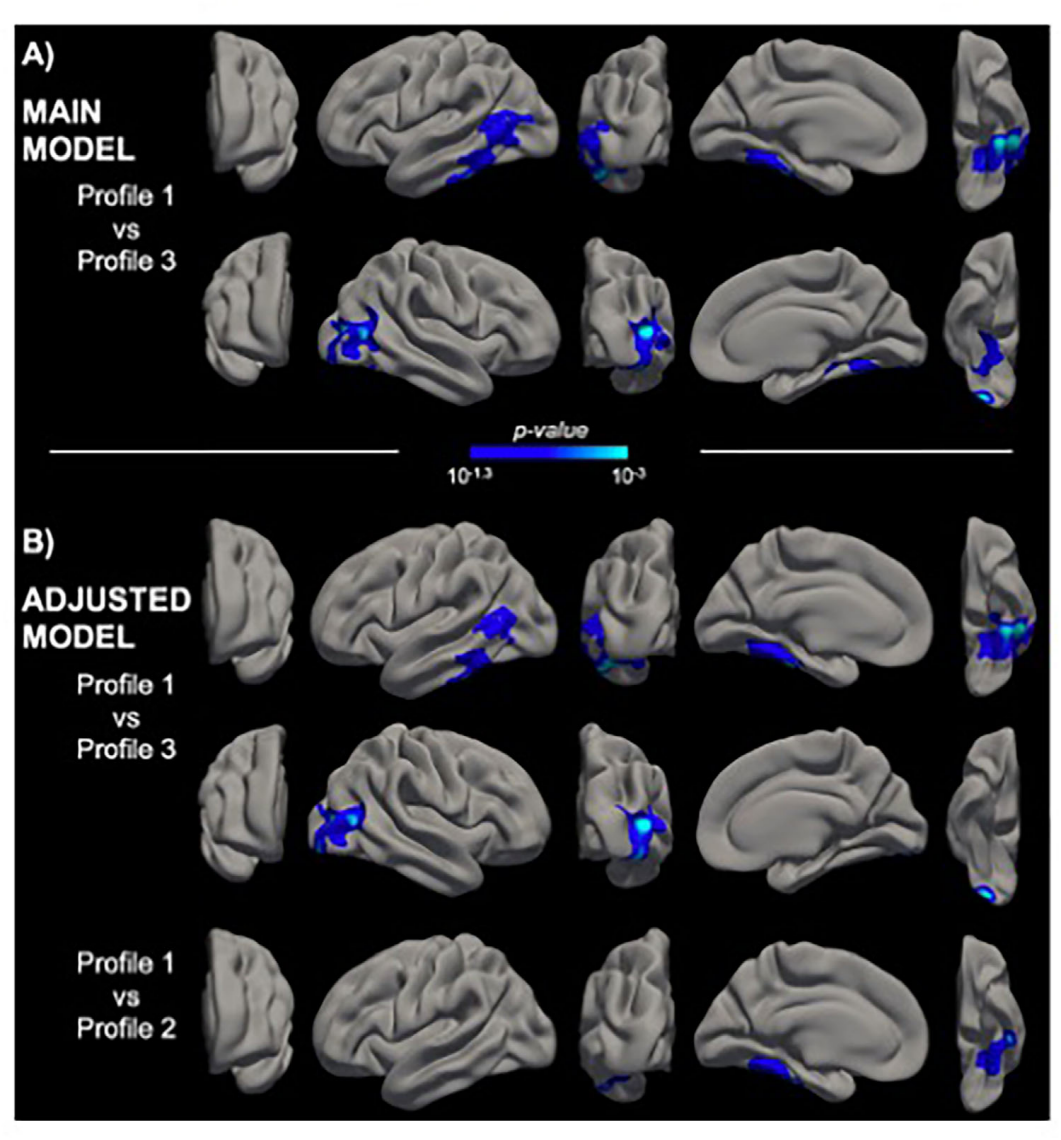# Psychological profiles associated with cortical thickness changes in middle‐aged adults

**DOI:** 10.1002/alz.091151

**Published:** 2025-01-03

**Authors:** David Bartrés‐Faz, Harriet Demnitz‐King, María Cabello‐Toscano, Lídia Vaqué‐Alcázar, Rob Saunders, Gabriele Cattaneo, Núria Bargalló, Josep M. Tormos‐Muñoz, Javier Solana Sánchez, Alvaro Pascual‐Leone, Natalie L. Marchant

**Affiliations:** ^1^ Department of Medicine, Faculty of Medicine and Health Sciences, Institute of Neurosciences, University of Barcelona, Barcelona, Spain. Institut d’Investigacions Biomèdiques August Pi i Sunyer (IDIBAPS), Barcelona Spain; ^2^ Guttmann Brain Health Institute, Institut Guttmann, Institut Universitari de Neurorehabilitació adscrit a la Universitat Autònoma de Barcelona, Barcelona Spain; ^3^ Institute of Biomedical Research August Pi i Sunyer (IDIBAPS), Barcelona Spain; ^4^ Division of Psychiatry, University College London, London United Kingdom; ^5^ Department of Medicine, Faculty of Medicine and Health Sciences, Institute of Neurosciences, University of Barcelona, Barcelona Spain; ^6^ Sant Pau Memory Unit, Hospital de la Santa Creu i Sant Pau, Biomedical Research Institute Sant Pau, Universitat Autònoma de Barcelona, Barcelona Spain; ^7^ University College London, London United Kingdom; ^8^ Institut Guttmann, Institut Universitari de Neurorehabilitació adscrit a la Universitat Autònoma de Barcelona, Badalona, Barcelona Spain; ^9^ Hospital Clinic, Barcelona Spain; ^10^ Centro de Investigación Translacional San Alberto Magno, Facultad Ciencias de la Salud, Universidad Católica de Valencia, Valencia Spain; ^11^ Institut Guttmann, Institut Universitari de Neurorehabilitació adscrit a la Universitat Autònoma de Barcelona, Barcelona Spain; ^12^ Hinda and Arthur Marcus Institute for Aging Research and Deanna and Sidney Wolk Center for Memory Health, Hebrew SeniorLife, Boston, MA USA

## Abstract

**Background:**

Psychological factors such as repetitive negative thinking, proneness to experience distress, and perceived stress are associated with increased risk of neurodegeneration and clinical dementia, whereas having a sense of life‐purpose, self‐reflection, and dispositional mindfulness may be protective. However, whether combinations of these risk and protective factors may inform distinct psychological profiles, which may be differential associated with age‐related health outcomes is currently unknown.

**Method:**

We included 742 middle‐aged (mean age 51.4 (SD:7.0), 51.1% women) healthy individuals from the Barcelona Brain Health Initiative. All participants underwent a psychological assessment of individual risk and protective factors based on previous literature (Figure 1). An initial 3T MRI was acquired for all participants, with follow‐up MRIs conducted for 533 participants (average follow‐up 2.3 years). Latent profile analysis (LPA) was used to identify statistically distinct psychological profiles from risk and protective factors and MRIs were automatically processed with FreeSurfer v6.0 (maps p < 0.05, corrected for family‐wise error).

**Result:**

LPA revealed a 3‐profile solution: Profile 1 (*n* = 190, 25.6%) was characterized by lower levels of positive or protective psychological factors; Profile 2 included individuals showing higher negative or psychological risk factors (*n* = 149, 20.1%); and Profile 3 reported high protective and moderately low risk factors (*n* = 403, 50.3%; Figure 1). Age‐, gender‐, and education‐adjusted results revealed that compared to Profile 3, individuals in Profile 1 exhibited accelerated cortical thinning in the inferior and middle temporal areas, including regions within the AD cortical thinning signature. In models additionally adjusted for anxiety and depressive symptoms, Profile 1 also exhibited greater cortical thinning over time in the inferior temporal lobe region compared to Profile 2 (Figure 2A and B).

**Conclusion:**

Using a person‐centered approach to identify statistically distinct psychological profiles we found that already in middle‐age, those with lower levels of protective factors (Profile 1) exhibit accelerated cortical atrophy. This emphasizes the need for comprehensive psychological assessments in dementia prevention research, including the assessment of ‘protective’ factors in addition to the more commonly assessed ‘risk’ factors.